# PD-1 and PD-L1 correlated gene expression profiles and their association with clinical outcomes of breast cancer

**DOI:** 10.1186/s12935-019-0955-2

**Published:** 2019-09-09

**Authors:** Cui Jiang, SunRun Cao, Na Li, Lei Jiang, Tao Sun

**Affiliations:** 1 Department of Medical Oncology, Cancer Hospital of China Medical University, Liaoning Cancer Hospital and & Institute, 44 Xiaoheyan Road, Dadong District, Shenyang, 110042 Liaoning People’s Republic of China; 2 Institute of Translational Medicine, China Medical University, No.77 Puhe Road, Shenyang, 110001 Liaoning People’s Republic of China

**Keywords:** PD-1, PD-L1, Immune, Breast cancer

## Abstract

**Background:**

Immunotherapies that targeting programmed cell death 1 (PD-1) and programmed death-ligand 1 (PD-L1) have obtained prominent success in breast cancer (BC). However, not all the patients benefit from the antibody therapy. This study aimed to identify PD-1/PD-L1 correlated genes and pathways as well as investigate their potential as prognostic marker in BC.

**Materials and methods:**

By analysing transcriptional data of BC from TCGA, we identified PD-1 and PD-L1 correlated genes by WGCNA analysis and explored the biological process as well as pathways they enriched. Co-expression analysis were performed for PD-1/PD-L1 with immune infiltration and checkpoints. The prognostic value of PD-1 and PD-L1 were also investigated.

**Results:**

PD-1 and PD-L1 expression showed significant difference in different molecular subtypes and stages. PD-1 correlated genes enriched in T cell activation, lymphocyte activation, leukocyte migration while PD-L1 correlated genes demonstrated enrichment including T cell apoptotic process, tolerance induction and cytolysis. Immune infiltration analysis suggested that PD-1 and PD-L1 were related with Neutrophils (r = 0.65, r = 0.48) and Fibroblasts (r = 0.59, r = 0.47). For immune checkpoints analysis, PD-1 was associated with HLA-A (r = 0.804) and INPP5D (r = 0.782) while PD-L1 correlated with CTLA4 (r = 0.843) and CD27 (r = 0.823). PD-1 was associated favorable survival of BC (HR = 0.67, P = 0.012) while PD-L1 did not demonstrate significant association with BC prognosis (HR = 0.85, P = 0.313).

**Conclusion:**

PD-1 and PD-L1 correlated genes participated in biological process including T cell activation, lymphocyte activation, leukocyte migration, T cell apoptotic process, tolerance induction and cytolysis. PD-1/PD-L1 expression also demonstrated relation with immune infiltration and immune checkpoints. High PD-1 expression predicted better survival of breast cancer patients.

## Background

As one of the most frequently occurred malignant tumors, breast cancer (BC) remains the leading cause of cancer-related death for females in many countries [1]. Breast cancer arises from multiple genetic factors, environmental alternations and their complicated interactions [2]. According to the status of biomarkers including estrogen receptor (ER), progesterone receptor (PR) and epidermal growth factor receptor 2 (HER2), patients with breast cancer were classified into groups of luminal A, luminal B, HER-2 positive and triple negative [3]. It has been accepted that different groups of BC patients benefit from corresponding treatment strategy of chemical and hormonal therapy [4].

Although certain therapeutic combinations have been used as standard treatment in clinical management of BC, some BC patients still could not get satisfactory clinical outcomes [5]. The different outcomes of BC patients indicated that other critical factors also determine the final therapeutic effect such as the immune status of the cells [6]. It is well-accepted that immune escape of tumor cells and aberrant human immune surveillance play essential role in carcinogenesis, progression and metastasis of various types of cancer [7]. As for immune escape of cancer cells, the identification of PD-1 (programmed death 1) and PD-L1 (programmed death-ligand 1) axis was one of the most encouraging finding of cancer therapy in recent years [8]. Serving as an immune checkpoint in tumor microenvironment, the antibodies of PD-1/PD-L1 has shown prominent effect in a large number of cancer types [9].

Previously, PD-L1 expression has been reported to be associated with worse prognosis of triple negative breast cancer patients, which counteract effect of tumor-infiltrating lymphocytes (TILs) [10]. Another study of HER2 + invasive BC patients indicated a positive correlation of PD-L1 expression and CD8+ T cells with favorable clinical outcomes [11]. One research of 1318 BC patients in European suggested that PD-1 positive immune cells in triple negative breast cancer correlated with longer disease-free survival, and tumor-infiltrating lymphocytes (TILs) density was remarkably related with PD-1 and PD-L1 expression in immune cells [12]. Most of the findings implied that medicine concerning PD-1 and PD-L1 immune checkpoint might become novel therapeutic strategies for breast cancer.

Although the critical role of immune checkpoint PD-1 and PD-L1 have been widely reported in a number of malignant tumors, the underlying regulating mechanisms in breast cancer is still unclear. In this study, we performed comprehensive analysis of gene expression profiles related to PD-1 and PD-L1 in breast cancer using transcriptome data from TCGA. The correlation of PD-1 and PD-L1 with other immune biomarkers and immune cells infiltration were revealed. Furthermore, the effect of PD-1 and PD-L1 on clinical outcomes of BC was explored to determine their potential as biomarkers for BC patients prognosis.

## Materials and methods

### Analyzed datasets

The RNA sequencing and clinical data of breast cancer patients in TCGA datasets were downloaded from UCSC XENA (https://xena.ucsc.edu/). The level of gene expression was measured as Transcripts per million reads (TPM). Clinical data included the histological type, molecular Type, cancer stage, recurrence event and survival information. The relationship between PD-1/PD-L1 expression and the clinical data were investigated.

### Co-expression gene and enrichment analysis

Weighted correlation network analysis (WGCNA) is an algorithm for finding genetic interactions in a weighted manner. Co-expressed genes obtained by WGCNA analysis will be more accurate. Using WGCNA analysis, we searched the co-expressed genes for PD-1 and PD-L1. As genes with little variation in expression usually represent noise, the most variant genes were filtered for network construction. Gene variabilities were measured by median absolute deviation (MAD). If the interaction gene was more than 200, we used the interaction degree to search the top 200 gene as the interaction genes. Clusterprofiler is a R package for enrichment analysis. Using clusterprofier, we used biological process in the Gene Ontology (GO) to analyze the interacted genes [13]. Since the results of the enrichment analysis contain many similar results, we further concentrated the results of the enrichment analysis.

### Relationship between immune factors and PD-1/PD-L1

A variety of studies have confirmed that immune infiltration and all aspects of the tumor are related. MCP-counter is available R package to estimate the sample immune infiltration. From a gene expression matrix, it produces for each sample an abundance score for CD3+ T cells, CD8+ T cells, cytotoxic lymphocytes, NK cells, B lymphocytes, cells originating from monocytes (monocytic lineage), myeloid dendritic cells, neutrophils, as well as endothelial cells and fibroblasts. Then we used correlation analysis to evaluate the correlation between PD-1/PD-L1 and immune infiltration. Immunological checkpoints serve as the primary site for detecting immune status, and we also evaluated the relationship between PD-1 and PD-L1 and immune checkpoints.

### Statistical analysis

In this study, statistical analysis was mainly performed by using R language (https://www.r-project.org/) with several publicly available packages. Rank sum test was used to evaluate the expression difference of PD-1/PD-L1 in different groups. Spearman correlation analysis was used to explore the correlation between PD-1/PD-L1 expression and immune infiltration and immune checkpoints. Survival curve was generated by Kaplan- Meier method based on log-rank test. Other Figures were generated by several R packages, such as pheatmap, circlize, and corrplot. All multiple tests were corrected by the BH method. A probability value P < 0.05 was considered to be significant in this study.

## Results

### PD-1/PD-L1 expression status in different clinical subgroups

**Fig. 1:**
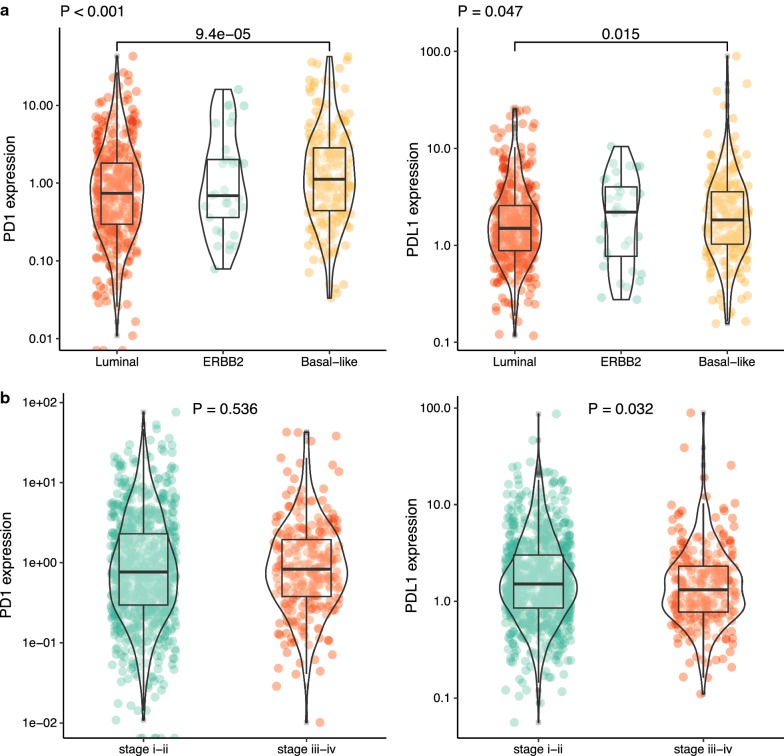
Differences in PD-1 and PD-L1 expression between different clinicopathological information. **a** PD-1 and PD-L1 expression in molecular type. **b** PD-1 and PD-L1 expression in stage

Using TCGA datasets, we analyzed the PD-1/PD-L1 expression in different groups according to the clinical data. As shown in Fig. 1a, both PD-1 and PD-L1 expression showed significant difference in the three molecular subtypes (P < 0.001 and P = 0.047, respectively). Luminal and Basal-like subtype show significant difference in PD-1 and PD-L1 expression. Moreover, the expression of PD-L1 differs among different stages (P = 0.032), while PD-1 did not show any difference (P = 0.536). In addition, both PD-1 and PD-L1 expression correlated with the recurrence event of BC patients (P = 0.017, P = 0.015, respectively). We also analyzed the relation of PD-1/PD-L1 expression with clinical data including therapy, histological subtype, ER, PR and HER-2 status (Table 1). Finally, both PD-1 and PD-L1 were correlated with ER, PR and clinical therapy, indicating the probable implication of PD-1/PD-L1 in clinical outcome.

**Table 1:** Association between PD-1/PD-L1 expression and clinical data

Group	Category	n	PD1 [mean (sd)]	PD1 P	PDL1 [mean (sd)]	PDL1 P
ER	Negative	212	4.35 (7.21)	< 0.001	4.35 (7.84)	< 0.001
ER	Positive	727	1.82 (3.55)		2.28 (3.19)	
Her2	Negative	495	2.54 (5.23)	0.27	3.04 (5.62)	0.39
Her2	Positive	152	2.04 (3.38)		2.62 (4.13)	
PR	Negative	308	3.47 (6.27)	< 0.001	3.41 (6.43)	0.003
PR	Positive	628	1.86 (3.68)		2.42 (3.59)	
Histological type	Infiltrating ductal carcinoma	790	2.48 (5.67)	0.274	2.94 (5.77)	0.278
Histological type	Infiltrating lobular carcinoma	205	2.58 (4.82)		2.36 (2.39)	
Histological type	Other type	107	1.62 (4.44)		2.42 (5.08)	
Therapy type	Chemotherapy	385	3.14 (6.96)	0.015	3.38 (6.38)	0.004
Therapy type	Hormone therapy	365	1.96 (4.24)		2.18 (2.82)	
Therapy type	Other therapy	31	2.02 (2.24)		2.56 (2.19)	

### Co-expression analysis of genes associated with PD-1 and PD-L1

**Fig. 2:**
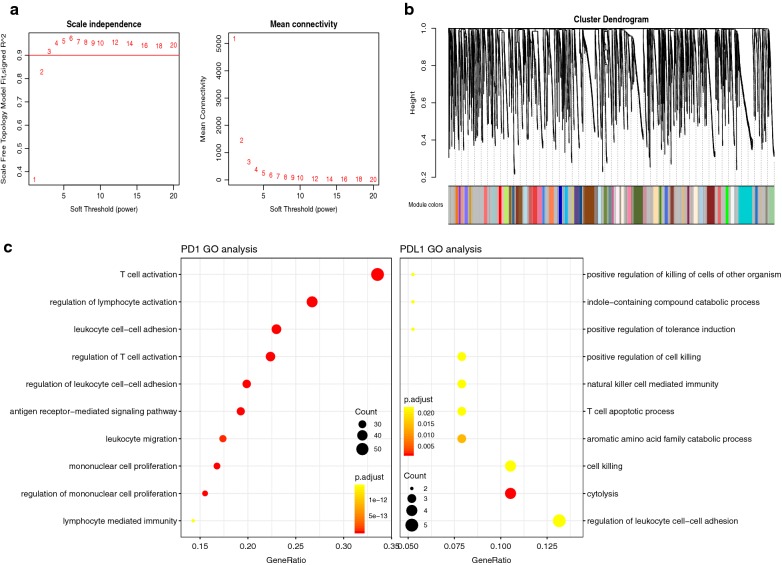
Co-expression analysis of genes associated with PD-1 and PD-L1. **a** Soft threshold selection in the WGCNA network analysis. **b** Gene distribution in the WGCNA network analysis. **c** GO analysis for the PD-1 and PD-L1 co-expression genes

**Fig. 3:**
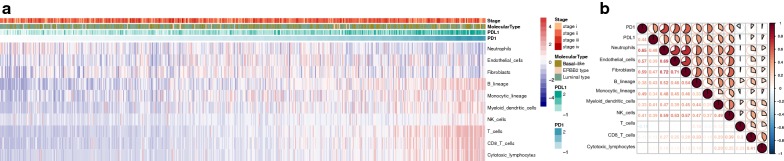
PD-1/PD-L1 expression and immune infiltration. **a** The proportion of all immune infiltration components in breast cancer. **b** co-expression analysis between PD-1/PD-L1 and immune infiltration

Using WGCNA, we analyzed the co-expression gene associated with PD-1 and PD-L1. The connectivity among genes was a scale-free network distribution if the value of soft thresholding power *β* equals to 3 (Fig. 2a). Altogether 21 module was obtained according to WGCNA analysis (Fig. 2b). Among these modules, PD-1 belonged to pink module while PD-L1 belonged to thistle 1 module. We finally got 1065 genes that interacted with PD-1 and 99 PD-L1 correlated genes. Then we selected the top 200 gene associated with PD-1 and all of the 99 PD-L1 related genes for further enrichment analysis. PD-1 correlated genes mainly enriched in biological process of T cell activation, regulation of lymphocyte activation, regulation of T cell activation and leukocyte migration while PD-L1 correlated genes demonstrated enrichment including positive regulation of killing of cells of other organism, T cell apoptotic process, positive regulation of tolerance induction and cytolysis (Fig. 3 and Table 2).

**Table 2:** Top ten terms of GO analysis for PD-1 and PD-L1

Gene	Description	GENERATIO	P value	P adjust	Count
PD1	T cell activation	54/161	8.67E−47	1.53E−43	54
	Regulation of lymphocyte activation	43/161	8.13E−32	7.17E−29	43
	Leukocyte cell–cell adhesion	37/161	9.69E−31	4.27E−28	37
	Regulation of T cell activation	36/161	1.45E−30	5.11E−28	36
	Antigen receptor-mediated signaling pathway	31/161	1.61E−26	4.74E−24	31
	Regulation of leukocyte cell–cell adhesion	32/161	4.29E−26	1.08E−23	32
	Regulation of mononuclear cell proliferation	25/161	1.22E−21	1.13E−19	25
	Mononuclear cell proliferation	27/161	3.08E−21	2.59E−19	27
	Leukocyte migration	28/161	1.13E−15	6.04E−14	28
	Lymphocyte mediated immunity	23/161	3.15E−14	1.43E−12	23
	Adaptive immune response	22/161	5.23E−13	2.15E−11	22
	Regulation of leukocyte mediated immunity	16/161	1.61E−11	5.57E−10	16
PDL1	Cytolysis	4/38	1.18E−06	0.00130442	4
	Aromatic amino acid family catabolic process	3/38	2.53E−05	0.01394493	3
	T cell apoptotic process	3/38	0.00014677	0.02200257	3
	Positive regulation of tolerance induction	2/38	0.0001831	0.02200257	2
	Indole-containing compound catabolic process	2/38	0.0001831	0.02200257	2
	Positive regulation of killing of cells of other organism	2/38	0.00026786	0.02200257	2
	Natural killer cell mediated immunity	3/38	0.00027843	0.02200257	3
	Cell killing	4/38	0.00030195	0.02200257	4
	Positive regulation of cell killing	3/38	0.00030586	0.02200257	3
	Regulation of leukocyte cell–cell adhesion	5/38	0.00031554	0.02200257	5
	Regulation of killing of cells of other organism	2/38	0.00036836	0.02200257	2
	Positive regulation of T cell apoptotic process	2/38	0.00036836	0.02200257	2

### PD-1/PD-L1 expression and immune infiltration

Using Microenvironment Cell Populations-counter, we evaluated the profiles of immune infiltration among various subtypes and stages breast cancer (Fig. 3a). Additionally, the associations of PD-1 and PD-L1 with immune cell populations according to the transcriptomic data were analyzed. The results indicated that PD-1 and PD-L1 were mainly related with Neutrophils (r = 0.65, r = 0.48) and Fibroblasts (r = 0.59, r = 0.47) (Fig. 3b).

### PD-1/PD-L1 expression and immune checkpoints

**Fig. 4:**
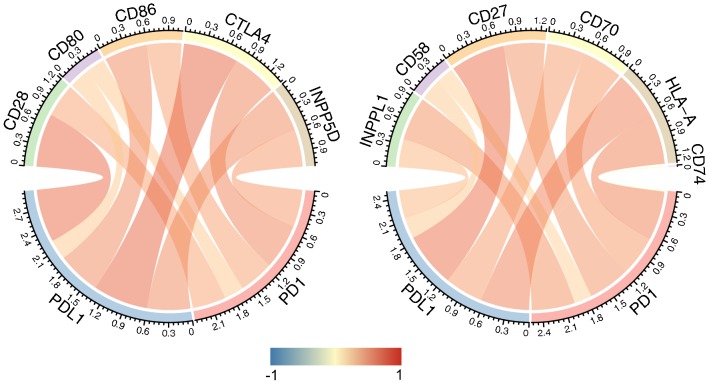
Co-expression analysis between PD-1/PD-L1 and immune checkpoints

As previous reported, the immune checkpoints mainly included CD28, CD80, CD86, CTLA4, INPP5D, INPPL1, CD58, CD27, CD70, HLA-A, CD74. We then analyzed the correlation between PD-1/PD-L1 expression and important immune checkpoints. As shown in Fig. 4 and Table 3, PD-1 was mainly associated with HLA-A (r = 0.804) and INPP5D (r = 0.782) while PD-L1 correlated with CTLA4 (r = 0.843) and CD27 (r = 0.823).

**Table 3:** Co-expression analysis between PD-1/PD-L1 and immune checkpoints

Gene	PD1_R	PD1_p	PD1_padj	PDL1_R	PDL1_p	PDL1_padj
CD28	0.68898768	3.05E−156	6.72E−156	0.72957675	4.77E−184	1.31E−183
CD80	0.50168845	1.98E−71	2.18E−71	0.46293539	9.94E−60	1.09E−59
CD86	0.61700599	8.49E−117	1.17E−116	0.70665274	9.35E−168	1.71E−167
CTLA4	0.7430565	1.96E−194	5.39E−194	0.84321417	2.52E−299	2.77E−298
INPP5D	0.78284443	2.58E−229	1.42E−228	0.6726487	2.64E−146	4.15E−146
INPPL1	0.6491299	4.48E−133	7.03E−133	0.61353869	3.75E−115	5.16E−115
CD58	0.61591686	2.80E−116	3.43E−116	0.59168023	3.10E−105	3.79E−105
CD27	0.74560817	1.79E−196	6.56E−196	0.82368263	9.05E−274	4.98E−273
CD70	0.68253159	3.08E−152	5.65E−152	0.72220769	1.24E−178	2.74E−178
HLA-A	0.80464345	9.84E−252	1.08E−250	0.74085677	1.08E−192	3.94E−192
CD74	0.1938974	8.19E−11	8.19E−11	0.16155305	6.77E−08	6.77E−08

P value was adjusted by ‘BH’ methods

### Survival analysis of PD-1/PD-L1 and PD-1/PD-L1 correlated genes

**Fig. 5:**
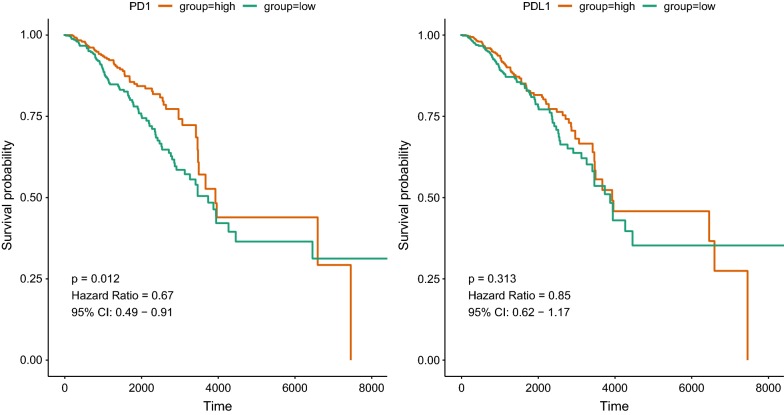
Prognostic analysis of PD-1/PD-L1

As PD-1/PD-L1 expression was correlated with clinical data, we then explored the prognostic value of PD-1/PD-L1 in breast cancer. As shown in Fig. 5 and Table 4, the expression of PD-1 was associated with favorable survival of breast cancer patients (HR = 0.67, 95% CI 0.49–0.91, P = 0.012) while PD-L1 did not demonstrate significant association with BC prognosis (HR = 0.85, 95% CI 0.62–1.17, P = 0.313). As for the multivariable analysis adjusting for age and stage, PD-1 still predicted better survival for BC patients (HR = 0.71, 95% CI 0.50–0.99, P = 0.045) but PD-L1 showed no significant relation (HR = 0.99, 95% CI 0.71–1.37, P = 0.934). In addition, PD-1/PD-L1 correlated genes were also analyzed in relation to prognosis of BC patients. The results suggested that certain immune markers interacting with PD-1/PD-L1 also correlated with survival of BC patients including CD5, CD74, CD96 and CD226 (Additional file 1: Table S1).

**Table 4:** Cox regression for PD-1 and PD-L1

Gene	HR (95% CI)	P	HR (95% CI) adjusted	P adjusted
PD1	0.67 (0.49 –0.91)	0.012	0.71 (0.50–0.99)	0.045
PDL1	0.85 (0.62 –1.17)	0.313	0.99 (0.71–1.37)	0.934

Adjust by stage and age

## Discussion

It is well-accepted that tumor and its microenvironment is a complex unit to complete the aggressive growth and metastasis of cancer cells [14–16]. Although a number of chemical and radical therapy have been used in clinical practice of cancer treatment, the immune system of human are still believed to be the most fundamental and effective weapon against cancer [17]. Programmed cell death 1 (PD-1) and corresponding ligand (PD-L1) are one of the most critical biological suppressors of cytotoxic immune reaction, the antibody of which has been authorized by FDA in a unprecedented fast term [18, 19]. However, the inhibitor of PD-1/PD-L1 were not effective in every individual, which require the comprehensive understanding of specific mechanisms underlying PD-1/PD-L1 regulation in carcinogenesis [20].

Using data from TCGA, we first unraveled the expression status of PD-1/PD-L1 in different subtypes and clinical stages of BC patients. PD-L1 expression was decreased in stage III-IV compared with stage I-II. On the basis of molecular classification, basal-like BC subtype showed highest expression of PD-1 and ERBB2 subtype BC had highest PD-L1 expression, which suggest that different subtypes possess various PD-1/PD-L1 status. In addition, PD-1 and PD-L1 expression correlated with the recurrence of BC patients, which also confirm the critical role of PD-1/PD-L1 immune checkpoint in BC progression.

We subsequently identified co-expressed genes of PD-1 and PD-L1 by means of WGCNA. A total of 1065 genes correlated with PD-1 and 99 PD-L1 correlated genes were screened. The GO enrichment analysis of PD-1 correlated genes suggested biological process of T cell activation, regulation of lymphocyte activation, regulation of T cell activation and leukocyte migration. In addition, PD-L1 correlated genes demonstrated enrichment including positive regulation of killing of cells of other organism, T cell apoptotic process, positive regulation of tolerance induction and cytolysis. The results of previous studies were in accordance with the GO enrichment of PD-1/PD-L1 related genes in functional modulation of T cell. For instance, one study suggested that during the transition from DCIS to an invasive lesion, the host cytolytic T cells interacted with the tumor and destroy the tumor tissue, leading to an adaptive upregulation of PD-L1 and tumor protection against immune destruction [21]. PD-L1 is expressed by antigen-presenting cells and results in T cell inactivation by interaction with PD-1 on T-cells. It is therefore of great importance to clarify the biological process and pathways we identified for PD-1/PDL1 related genes, by which immunotherapy with PD-1- and PD-L1-targeted monoclonal antibodies might dramatically change the therapeutic and prognostic landscape for cancer.

Immune infiltration of breast cancer determine the immune activation of tumor microenvironment and is related with clinical outcome of patients. According to the correlation analysis of PD-1 and PD-L1 with immune cell populations, PD-1 and PD-L1 were mainly related with Neutrophils and Fibroblasts. It has been reported that PD-1 protein expression significantly correlated with higher TIL abundance, Ki-67 index, basal-like subtypes, and distant metastasis of triple-negative breast cancer (TNBC) [22]. The interaction of PD-1/PD-L1 immune checkpoint with immune infiltration is a promising research direction in the future. The immune checkpoints of CD28, CD80, CD86, CTLA4, INPP5D, INPPL1, CD58, CD27, CD70, HLA-A, CD74 were also analyzed in relation to PD-1/PD-L1 expression. Finally, PD-1 was found to be mainly associated with HLA-A and INPP5D while PD-L1 correlated with CTLA4 and CD27. Cytotoxic T-lymphocyte-associated protein 4 (CTLA4) belongs to immunoglobulin superfamily and encodes a protein transmitting inhibitory signal to T cells, the antibody of which also demonstrated favorable outcome in clinical management [23]. The combination usage of PD-1/PD-L1 with these immune checkpoints might become novel therapeutic targets in the future [24].

Survival analysis of PD-1/PD-L1 expression status demonstrated that the expression of PD-1 was associated favorable survival of breast cancer patients while PD-L1 did not suggest significant association with BC prognosis. In a study of 195 triple-negative breast cancer individuals, PD-1 was found to be significantly related with better disease free survival and overall survival. The results of this study also suggested that PD-1 protein expression in TILs, but not PD-L1 in tumor cells, predicted better prognosis in TNBC [22]. Researchers demonstrated that high expression of PDL1 are associated with favorable clinical outcome in 127 primary breast cancer [25]. Another study of HER2+ invasive BC patients indicated a positive correlation of PD-L1 expression and CD8+ T cells with favorable clinical outcomes [11]. On contrary, PD-L1 expression has been reported to be associated with worse prognosis of triple negative breast cancer patients, which counteract effect of tumor-infiltrating lymphocytes [10]. Response of BC patients to neoadjuvant therapy and survival outcome indicated that PD-L1 predicted better rate of pathological complete response (pCR) [26]. In addition, PD-1/PD-L1 correlated genes such as CD5, CD74, CD96 and CD226 were also related with prognosis of BC patients. The combination of these immune markers with PD-1/PD-L1 might improve the prediction and management of BC patients in the future. Until now, whether PD-1/PL-L1 predict prognosis in BC patients was still in debate. The specific association of PD-1/PD-L1 with BC prognosis still required further large-scale investigations to confirm, which would benefit the potential of PD-1/PD-L1 as prognostic biomarker.

## Conclusion

In this study, we reported the expression status of PD-1 and PD-L1 in different subtypes of breast cancer. The PD-1/PDL1 correlated gene profiles were described, the enrichment analysis of which focus on biological process including T cell activation, lymphocyte activation, leukocyte migration, T cell apoptotic process, tolerance induction and cytolysis. PD-1/PD-L1 expression also demonstrated relation with immune cells infiltration and multiple immune checkpoints. High PD-1 expression was associated with better survival of breast cancer patients.

## Supplementary information

**Additional file 1: Table S1.** Prognostic analysis of PD1/PDL1 co-expressing genes.

## Data Availability

The data that support the findings of this study are available from TCGA.

## Abbreviations

PD-1: programmed cell death 1
PD-L1: programmed death-ligand 1
BC: breast cancer
ER: estrogen receptor
PR: progesterone receptor
HER2: epidermal growth factor receptor 2
TILs: tumor-infiltrating lymphocytes
TPM: transcripts per million reads
WGCNA: weighted correlation network analysis
MAD: median absolute deviation
GO: Gene Ontology
Monocytic lineage: cells originating from monocytes
TNBC: triple-negative breast cancer
CTLA4: cytotoxic T-lymphocyte-associated protein 4
pCR: pathological complete response
