# Supplementary material for: PD-1 and PD-L1 correlated gene expression profiles and their association with clinical outcomes of breast cancer
Source: Cancer Cell Int. 2019 Sep 9;19:233. doi: 10.1186/s12935-019-0955-2 (PMC6734479; doi:10.1186/s12935-019-0955-2)
Supplement: Supplementary file 1 — Additional file 1: Table S1. Prognostic analysis of PD1/PDL1 co-expressing genes. [file 12935_2019_955_MOESM1_ESM.docx]

Additional file 1: Table S1. Prognostic analysis of PD1/PDL1 co-expressing genes

| gene | ensid | gsm | HR | low95 | up95 | p.val |
| --- | --- | --- | --- | --- | --- | --- |
| PD-1 | ENSG00000111796.3 | KLRB1 | 0.508242 | 0.371111 | 0.696044 | 2.87E-05 |
| PD-1 | ENSG00000138378.16 | STAT4 | 0.550287 | 0.402052 | 0.753174 | 0.00025 |
| PD-1 | ENSG00000172543.6 | CTSW | 0.558901 | 0.408299 | 0.765055 | 0.000341 |
| PD-1 | ENSG00000019582.13 | CD74 | 0.573124 | 0.418531 | 0.78482 | 0.000561 |
| PD-1 | ENSG00000128340.13 | RAC2 | 0.57496 | 0.419774 | 0.787518 | 0.000595 |
| PD-1 | ENSG00000167664.7 | TMIGD2 | 0.578075 | 0.422177 | 0.791541 | 0.000667 |
| PD-1 | ENSG00000135426.13 | TESPA1 | 0.58508 | 0.427333 | 0.801058 | 0.000823 |
| PD-1 | ENSG00000240505.7 | TNFRSF13B | 0.581723 | 0.424939 | 0.796352 | 0.000826 |
| PD-1 | ENSG00000015285.9 | WAS | 0.585437 | 0.427714 | 0.80132 | 0.00096 |
| PD-1 | ENSG00000102245.6 | CD40LG | 0.594451 | 0.434316 | 0.813629 | 0.001296 |
| PD-1 | ENSG00000198851.8 | CD3E | 0.598569 | 0.437249 | 0.819407 | 0.001448 |
| PD-1 | ENSG00000175463.10 | TBC1D10C | 0.597059 | 0.436208 | 0.817223 | 0.001523 |
| PD-1 | ENSG00000069493.13 | CLEC2D | 0.607689 | 0.443679 | 0.832328 | 0.001903 |
| PD-1 | ENSG00000183918.13 | SH2D1A | 0.608591 | 0.444606 | 0.833059 | 0.002019 |
| PD-1 | ENSG00000153283.11 | CD96 | 0.608605 | 0.444654 | 0.833007 | 0.00208 |
| PD-L1 | ENSG00000111537.4 | IFNG | 0.609672 | 0.445338 | 0.834648 | 0.002179 |
| PD-1 | ENSG00000160185.12 | UBASH3A | 0.610391 | 0.445926 | 0.835512 | 0.002252 |
| PD-1 | ENSG00000145287.9 | PLAC8 | 0.612226 | 0.447132 | 0.838278 | 0.002282 |
| PD-L1 | ENSG00000100721.9 | TCL1A | 0.612334 | 0.447294 | 0.83827 | 0.002288 |
| PD-1 | ENSG00000239713.6 | APOBEC3G | 0.613572 | 0.448018 | 0.840303 | 0.002301 |
| PD-1 | ENSG00000113263.11 | ITK | 0.614396 | 0.448809 | 0.841077 | 0.00235 |
| PD-1 | ENSG00000174946.6 | GPR171 | 0.615308 | 0.449478 | 0.842317 | 0.002545 |
| PD-1 | ENSG00000035720.6 | STAP1 | 0.616936 | 0.450674 | 0.844534 | 0.002721 |
| PD-1 | ENSG00000188822.7 | CNR2 | 0.618508 | 0.451828 | 0.846675 | 0.002888 |
| PD-1 | ENSG00000105369.8 | CD79A | 0.618338 | 0.45163 | 0.846583 | 0.00289 |
| PD-1 | ENSG00000138755.5 | CXCL9 | 0.621863 | 0.454335 | 0.851165 | 0.003259 |
| PD-L1 | ENSG00000090554.11 | FLT3LG | 0.619974 | 0.452967 | 0.848558 | 0.003338 |
| PD-1 | ENSG00000110448.9 | CD5 | 0.625794 | 0.457213 | 0.856533 | 0.003585 |
| PD-1 | ENSG00000150637.7 | CD226 | 0.625219 | 0.456792 | 0.855749 | 0.003691 |
| PD-1 | ENSG00000005844.16 | ITGAL | 0.628311 | 0.458962 | 0.860146 | 0.003962 |
| PD-1 | ENSG00000271856.1 | LINC01215 | 0.630392 | 0.460548 | 0.862874 | 0.004143 |
| PD-1 | ENSG00000073861.2 | TBX21 | 0.630718 | 0.460799 | 0.863294 | 0.004465 |
| PD-1 | ENSG00000167286.8 | CD3D | 0.632399 | 0.462047 | 0.865558 | 0.004545 |
| PD-1 | ENSG00000198821.9 | CD247 | 0.633929 | 0.46314 | 0.867698 | 0.00455 |
| PD-1 | ENSG00000116824.4 | CD2 | 0.633921 | 0.463134 | 0.867688 | 0.004654 |
| PD-1 | ENSG00000238121.4 | LINC00426 | 0.63357 | 0.462903 | 0.867159 | 0.00473 |
| PD-1 | ENSG00000145649.7 | GZMA | 0.635412 | 0.464251 | 0.869679 | 0.004895 |
| PD-1 | ENSG00000013725.13 | CD6 | 0.635965 | 0.464655 | 0.870435 | 0.004915 |
| PD-1 | ENSG00000163519.12 | TRAT1 | 0.638399 | 0.466415 | 0.873799 | 0.005096 |
| PD-1 | ENSG00000125910.5 | S1PR4 | 0.635473 | 0.464262 | 0.869824 | 0.005143 |
| PD-1 | ENSG00000023892.10 | DEF6 | 0.639614 | 0.467305 | 0.875458 | 0.005802 |
| PD-1 | ENSG00000177455.10 | CD19 | 0.642237 | 0.46903 | 0.879405 | 0.005844 |
| PD-1 | ENSG00000009790.13 | TRAF3IP3 | 0.641085 | 0.468346 | 0.877534 | 0.006131 |
| PD-1 | ENSG00000077984.5 | CST7 | 0.645847 | 0.471868 | 0.883972 | 0.00643 |
| PD-1 | ENSG00000146285.12 | SCML4 | 0.647438 | 0.473033 | 0.886147 | 0.006725 |
| PD-1 | ENSG00000132185.15 | FCRLA | 0.64683 | 0.472496 | 0.885486 | 0.006816 |
| PD-1 | ENSG00000186810.7 | CXCR3 | 0.651306 | 0.475862 | 0.891434 | 0.007663 |
| PD-1 | ENSG00000172116.20 | CD8B | 0.651667 | 0.476049 | 0.892072 | 0.007836 |
| PD-1 | ENSG00000172724.10 | CCL19 | 0.65502 | 0.478215 | 0.897193 | 0.008265 |
| PD-L1 | ENSG00000180644.6 | PRF1 | 0.652008 | 0.476288 | 0.892558 | 0.008329 |
| PD-1 | ENSG00000236320.3 | SLFN14 | 0.654897 | 0.478481 | 0.896356 | 0.008727 |
| PD-L1 | ENSG00000065675.13 | PRKCQ | 0.661001 | 0.482937 | 0.904719 | 0.009735 |
| PD-L1 | ENSG00000131203.11 | IDO1 | 0.660585 | 0.482529 | 0.904345 | 0.009749 |
| PD-1 | ENSG00000247774.5 | PCED1B-AS1 | 0.660983 | 0.482924 | 0.904695 | 0.010087 |
| PD-L1 | ENSG00000147168.11 | IL2RG | 0.661962 | 0.48364 | 0.906032 | 0.010112 |
| PD-1 | ENSG00000089012.13 | SIRPG | 0.661639 | 0.483414 | 0.905571 | 0.010289 |
| PD-1 | ENSG00000100351.15 | GRAP2 | 0.662184 | 0.483803 | 0.906335 | 0.01034 |
| PD-1 | ENSG00000205045.7 | SLFN12L | 0.662298 | 0.483896 | 0.906473 | 0.010433 |
| PD-1 | ENSG00000113088.5 | GZMK | 0.664912 | 0.485805 | 0.910053 | 0.010562 |
| PD-1 | ENSG00000139193.3 | CD27 | 0.663428 | 0.484679 | 0.9081 | 0.010777 |
| PD-1 | ENSG00000281103.1 | TRG-AS1 | 0.66493 | 0.485818 | 0.910077 | 0.011134 |
| PD-1 | ENSG00000197540.6 | GZMM | 0.665506 | 0.486215 | 0.910909 | 0.011143 |
| PD-1 | ENSG00000172349.15 | IL16 | 0.666908 | 0.487259 | 0.912791 | 0.011331 |
| PD-L1 | ENSG00000269404.5 | SPIB | 0.665722 | 0.486303 | 0.911336 | 0.011333 |
| PD-1 | ENSG00000101082.12 | SLA2 | 0.667178 | 0.487429 | 0.913215 | 0.011592 |
| PD-1 | ENSG00000104814.11 | MAP4K1 | 0.669343 | 0.48904 | 0.91612 | 0.012507 |
| PD-1 | ENSG00000115085.12 | ZAP70 | 0.669723 | 0.489319 | 0.91664 | 0.012509 |
| PD-1 | ENSG00000140368.11 | PSTPIP1 | 0.667628 | 0.487761 | 0.913822 | 0.01266 |
| PD-1 | ENSG00000163564.13 | PYHIN1 | 0.670454 | 0.489853 | 0.917639 | 0.012774 |
| PD-1 | ENSG00000188389.9 | PDCD1 | 0.66988 | 0.489431 | 0.916859 | 0.012877 |
| PD-1 | ENSG00000255733.4 | IFNG-AS1 | 0.672862 | 0.491509 | 0.921128 | 0.013703 |
| PD-L1 | ENSG00000100453.11 | GZMB | 0.672014 | 0.490988 | 0.919783 | 0.01402 |
| PD-1 | ENSG00000102879.14 | CORO1A | 0.673497 | 0.492035 | 0.921883 | 0.014731 |
| PD-1 | ENSG00000105374.8 | NKG7 | 0.677965 | 0.495343 | 0.927916 | 0.016022 |
| PD-1 | ENSG00000171608.14 | PIK3CD | 0.678902 | 0.496027 | 0.929198 | 0.016204 |
| PD-L1 | ENSG00000086730.15 | LAT2 | 0.677137 | 0.494514 | 0.927201 | 0.016493 |
| PD-1 | ENSG00000163600.11 | ICOS | 0.681746 | 0.498098 | 0.933106 | 0.017531 |
| PD-L1 | ENSG00000100298.14 | APOBEC3H | 0.684205 | 0.499865 | 0.936524 | 0.017992 |
| PD-L1 | ENSG00000229205.2 | LINC00200 | 1.485342 | 1.034722 | 2.132206 | 0.018488 |
| PD-1 | ENSG00000164483.15 | SAMD3 | 0.685688 | 0.500981 | 0.938494 | 0.018518 |
| PD-1 | ENSG00000181847.10 | TIGIT | 0.686334 | 0.501454 | 0.939377 | 0.018948 |
| PD-1 | ENSG00000162739.12 | SLAMF6 | 0.688538 | 0.503041 | 0.942436 | 0.019451 |
| PD-1 | ENSG00000028137.15 | TNFRSF1B | 0.687613 | 0.502388 | 0.941129 | 0.019595 |
| PD-1 | ENSG00000007312.11 | CD79B | 0.690773 | 0.504323 | 0.946154 | 0.020512 |
| PD-1 | ENSG00000163219.10 | ARHGAP25 | 0.690335 | 0.504374 | 0.944859 | 0.021068 |
| PD-L1 | ENSG00000077150.16 | NFKB2 | 0.692358 | 0.50562 | 0.948064 | 0.021622 |
| PD-1 | ENSG00000182866.15 | LCK | 0.693289 | 0.506539 | 0.94889 | 0.022159 |
| PD-1 | ENSG00000137078.7 | SIT1 | 0.692329 | 0.505828 | 0.947593 | 0.022262 |
| PD-1 | ENSG00000023445.12 | BIRC3 | 0.694239 | 0.507102 | 0.950434 | 0.022628 |
| PD-1 | ENSG00000075884.11 | ARHGAP15 | 0.695686 | 0.508276 | 0.952197 | 0.023491 |
| PD-1 | ENSG00000117091.8 | CD48 | 0.696664 | 0.509005 | 0.953508 | 0.023652 |
| PD-1 | ENSG00000167208.13 | SNX20 | 0.696862 | 0.509089 | 0.953892 | 0.024528 |
| PD-L1 | ENSG00000108984.12 | MAP2K6 | 0.697859 | 0.50967 | 0.955533 | 0.024578 |
| PD-L1 | ENSG00000081059.18 | TCF7 | 0.70009 | 0.511052 | 0.959052 | 0.025396 |
| PD-1 | ENSG00000235532.1 | LINC00402 | 0.700081 | 0.511494 | 0.958201 | 0.025528 |
| PD-1 | ENSG00000153563.14 | CD8A | 0.700443 | 0.511759 | 0.958696 | 0.026002 |
| PD-1 | ENSG00000164691.15 | TAGAP | 0.702027 | 0.512922 | 0.960852 | 0.027035 |
| PD-1 | ENSG00000163508.11 | EOMES | 0.701262 | 0.512289 | 0.959943 | 0.027398 |
| PD-1 | ENSG00000124203.5 | ZNF831 | 0.704632 | 0.514739 | 0.96458 | 0.028106 |
| PD-1 | ENSG00000185811.15 | IKZF1 | 0.706687 | 0.5163 | 0.967279 | 0.029602 |
| PD-1 | ENSG00000180096.10 | 1-Sep | 0.704907 | 0.514996 | 0.96485 | 0.029774 |
| PD-L1 | ENSG00000188676.12 | IDO2 | 0.706185 | 0.515879 | 0.966695 | 0.029873 |
| PD-L1 | ENSG00000027869.10 | SH2D2A | 0.70845 | 0.517224 | 0.970373 | 0.030948 |
| PD-1 | ENSG00000020633.17 | RUNX3 | 0.707659 | 0.516961 | 0.968703 | 0.030995 |
| PD-1 | ENSG00000149781.11 | FERMT3 | 0.706857 | 0.516146 | 0.968035 | 0.032179 |
| PD-L1 | ENSG00000104432.11 | IL7 | 0.708668 | 0.517752 | 0.969983 | 0.032348 |
| PD-1 | ENSG00000077420.14 | APBB1IP | 0.711315 | 0.519699 | 0.97358 | 0.033546 |
| PD-L1 | ENSG00000125735.9 | TNFSF14 | 0.712924 | 0.520885 | 0.975764 | 0.035268 |
| PD-L1 | ENSG00000100450.11 | GZMH | 0.712573 | 0.520577 | 0.975381 | 0.035848 |
| PD-1 | ENSG00000173200.11 | PARP15 | 0.714107 | 0.521735 | 0.977409 | 0.035964 |
| PD-1 | ENSG00000197471.10 | SPN | 0.716134 | 0.523167 | 0.980276 | 0.036572 |
| PD-1 | ENSG00000173762.6 | CD7 | 0.715775 | 0.522952 | 0.979698 | 0.037349 |
| PD-1 | ENSG00000110665.10 | C11orf21 | 0.715951 | 0.523034 | 0.980024 | 0.037851 |
| PD-1 | ENSG00000160654.8 | CD3G | 0.718131 | 0.524689 | 0.982891 | 0.038654 |
| PD-1 | ENSG00000056558.9 | TRAF1 | 0.720424 | 0.526263 | 0.986218 | 0.040581 |
| PD-1 | ENSG00000186265.8 | BTLA | 0.72306 | 0.528257 | 0.9897 | 0.041855 |
| PD-L1 | ENSG00000122223.11 | CD244 | 0.72369 | 0.528661 | 0.990669 | 0.044955 |
| PD-1 | ENSG00000122122.9 | SASH3 | 0.725773 | 0.530083 | 0.993706 | 0.045856 |
| PD-1 | ENSG00000105122.11 | RASAL3 | 0.725232 | 0.529781 | 0.992791 | 0.045972 |
| PD-L1 | ENSG00000117560.7 | FASLG | 0.726907 | 0.531095 | 0.994914 | 0.046981 |
| PD-L1 | ENSG00000090104.10 | RGS1 | 0.731788 | 0.534532 | 1.001836 | 0.047827 |
| PD-1 | ENSG00000123329.16 | ARHGAP9 | 0.728348 | 0.532045 | 0.997079 | 0.048964 |
| PD-1 | ENSG00000269220.1 | LINC00528 | 0.728672 | 0.53239 | 0.997318 | 0.049024 |
| PD-1 | ENSG00000043462.10 | LCP2 | 0.730156 | 0.533358 | 0.999569 | 0.049447 |
| PD-1 | ENSG00000198286.8 | CARD11 | 0.729303 | 0.532641 | 0.998577 | 0.050188 |
| PD-1 | ENSG00000179144.4 | GIMAP7 | 0.731904 | 0.534515 | 1.002185 | 0.050631 |
| PD-1 | ENSG00000138964.15 | PARVG | 0.731213 | 0.534025 | 1.001213 | 0.052834 |
| PD-L1 | ENSG00000100079.6 | LGALS2 | 0.734793 | 0.53681 | 1.005795 | 0.053588 |
| PD-1 | ENSG00000160856.19 | FCRL3 | 0.734838 | 0.536884 | 1.00578 | 0.053861 |
| PD-1 | ENSG00000130755.11 | GMFG | 0.735414 | 0.537317 | 1.006545 | 0.054679 |
| PD-1 | ENSG00000076662.8 | ICAM3 | 0.73601 | 0.537702 | 1.007454 | 0.055351 |
| PD-1 | ENSG00000136286.13 | MYO1G | 0.733814 | 0.535909 | 1.004804 | 0.055354 |
| PD-1 | ENSG00000028277.19 | POU2F2 | 0.734564 | 0.536558 | 1.005641 | 0.055369 |
| PD-1 | ENSG00000115165.8 | CYTIP | 0.740299 | 0.540884 | 1.013235 | 0.057394 |
| PD-1 | ENSG00000233308.1 | OSTN-AS1 | 0.73924 | 0.540111 | 1.011785 | 0.060133 |
| PD-1 | ENSG00000012124.13 | CD22 | 0.739776 | 0.540464 | 1.012591 | 0.0602 |
| PD-1 | ENSG00000254838.5 | GVINP1 | 0.740449 | 0.540968 | 1.013488 | 0.060479 |
| PD-1 | ENSG00000167895.13 | TMC8 | 0.741494 | 0.54173 | 1.014922 | 0.061366 |
| PD-L1 | ENSG00000100342.19 | APOL1 | 0.741761 | 0.54195 | 1.015241 | 0.061992 |
| PD-1 | ENSG00000166501.11 | PRKCB | 0.743677 | 0.543351 | 1.017861 | 0.062544 |
| PD-L1 | ENSG00000055208.16 | TAB2 | 1.347418 | 0.984459 | 1.844196 | 0.063188 |
| PD-1 | ENSG00000023902.12 | PLEKHO1 | 0.742512 | 0.542499 | 1.016267 | 0.064169 |
| PD-1 | ENSG00000147138.1 | GPR174 | 0.74658 | 0.54545 | 1.021876 | 0.068442 |
| PD-1 | ENSG00000185905.3 | C16orf54 | 0.748624 | 0.546968 | 1.024626 | 0.068658 |
| PD-1 | ENSG00000104894.10 | CD37 | 0.747539 | 0.546065 | 1.023348 | 0.0693 |
| PD-1 | ENSG00000100055.19 | CYTH4 | 0.746031 | 0.544593 | 1.021979 | 0.070281 |
| PD-L1 | ENSG00000004468.11 | CD38 | 0.749144 | 0.547348 | 1.025338 | 0.071796 |
| PD-1 | ENSG00000123338.11 | NCKAP1L | 0.751017 | 0.548591 | 1.028137 | 0.072881 |
| PD-1 | ENSG00000160219.10 | GAB3 | 0.754041 | 0.550786 | 1.032302 | 0.077055 |
| PD-L1 | ENSG00000065357.18 | DGKA | 0.753046 | 0.5502 | 1.030678 | 0.077549 |
| PD-1 | ENSG00000161929.13 | SCIMP | 0.7555 | 0.551845 | 1.034312 | 0.078635 |
| PD-1 | ENSG00000109943.7 | CRTAM | 0.755882 | 0.552252 | 1.034596 | 0.079912 |
| PD-L1 | ENSG00000104921.13 | FCER2 | 0.755657 | 0.552107 | 1.034252 | 0.080057 |
| PD-L1 | ENSG00000233610.1 | LINC00462 | 0.755781 | 0.552187 | 1.034441 | 0.080514 |
| PD-1 | ENSG00000117090.13 | SLAMF1 | 0.757488 | 0.553444 | 1.036759 | 0.082015 |
| PD-L1 | ENSG00000181873.11 | IBA57 | 1.318896 | 0.962481 | 1.807295 | 0.082571 |
| PD-1 | ENSG00000133574.8 | GIMAP4 | 0.759026 | 0.554562 | 1.038875 | 0.084296 |
| PD-1 | ENSG00000235304.1 | LINC01281 | 0.758795 | 0.554293 | 1.038747 | 0.084962 |
| PD-1 | ENSG00000145779.7 | TNFAIP8 | 0.764028 | 0.558193 | 1.045766 | 0.090464 |
| PD-1 | ENSG00000226979.7 | LTA | 0.762336 | 0.556954 | 1.043454 | 0.090823 |
| PD-1 | ENSG00000143119.11 | CD53 | 0.764313 | 0.558331 | 1.046286 | 0.091494 |
| PD-L1 | ENSG00000074966.9 | TXK | 0.767366 | 0.560654 | 1.050292 | 0.095134 |
| PD-1 | ENSG00000178199.12 | ZC3H12D | 0.767387 | 0.560564 | 1.050517 | 0.099405 |
| PD-1 | ENSG00000081237.17 | PTPRC | 0.769323 | 0.56197 | 1.053183 | 0.101114 |
| PD-L1 | ENSG00000096996.14 | IL12RB1 | 0.769538 | 0.562024 | 1.053671 | 0.102473 |
| PD-1 | ENSG00000161405.15 | IKZF3 | 0.772083 | 0.564093 | 1.056763 | 0.105272 |
| PD-1 | ENSG00000180353.9 | HCLS1 | 0.77321 | 0.56487 | 1.058392 | 0.105991 |
| PD-1 | ENSG00000176083.16 | ZNF683 | 0.771947 | 0.563995 | 1.056573 | 0.106274 |
| PD-1 | ENSG00000010810.16 | FYN | 0.773304 | 0.565001 | 1.058404 | 0.106932 |
| PD-L1 | ENSG00000105246.5 | EBI3 | 0.773608 | 0.565092 | 1.059065 | 0.107969 |
| PD-1 | ENSG00000213654.8 | GPSM3 | 0.774603 | 0.565883 | 1.060306 | 0.108149 |
| PD-L1 | ENSG00000259803.5 | SLC22A31 | 0.773118 | 0.564849 | 1.05818 | 0.109481 |
| PD-1 | ENSG00000143185.3 | XCL2 | 0.776528 | 0.567346 | 1.062835 | 0.113378 |
| PD-1 | ENSG00000163599.13 | CTLA4 | 0.776393 | 0.567237 | 1.062672 | 0.113988 |
| PD-1 | ENSG00000012779.9 | ALOX5 | 0.778136 | 0.568251 | 1.065543 | 0.116596 |
| PD-L1 | ENSG00000026751.15 | SLAMF7 | 0.778167 | 0.56839 | 1.065367 | 0.118582 |
| PD-L1 | ENSG00000078589.11 | P2RY10 | 0.781498 | 0.57095 | 1.069688 | 0.122312 |
| PD-1 | ENSG00000110876.9 | SELPLG | 0.783756 | 0.572444 | 1.073073 | 0.126485 |
| PD-1 | ENSG00000178562.16 | CD28 | 0.784503 | 0.573081 | 1.073924 | 0.126539 |
| PD-1 | ENSG00000143851.14 | PTPN7 | 0.784852 | 0.573432 | 1.07422 | 0.127585 |
| PD-1 | ENSG00000110934.9 | BIN2 | 0.78446 | 0.573049 | 1.073864 | 0.129063 |
| PD-L1 | ENSG00000101017.12 | CD40 | 0.784817 | 0.573407 | 1.074172 | 0.129642 |
| PD-1 | ENSG00000105639.17 | JAK3 | 0.785618 | 0.573995 | 1.075261 | 0.129983 |
| PD-L1 | ENSG00000025708.11 | TYMP | 0.786375 | 0.574441 | 1.0765 | 0.136495 |
| PD-L1 | ENSG00000142512.13 | SIGLEC10 | 0.791522 | 0.578179 | 1.083587 | 0.143719 |
| PD-1 | ENSG00000131401.10 | NAPSB | 0.793702 | 0.579904 | 1.086323 | 0.14801 |
| PD-1 | ENSG00000174255.6 | ZNF80 | 0.79556 | 0.58125 | 1.088888 | 0.150597 |
| PD-1 | ENSG00000117215.13 | PLA2G2D | 0.794583 | 0.580548 | 1.087528 | 0.151017 |
| PD-1 | ENSG00000182162.8 | P2RY8 | 0.796481 | 0.581914 | 1.090165 | 0.152085 |
| PD-1 | ENSG00000168918.12 | INPP5D | 0.796736 | 0.58212 | 1.090475 | 0.15581 |
| PD-L1 | ENSG00000092345.12 | DAZL | 0.797466 | 0.582645 | 1.091493 | 0.156826 |
| PD-1 | ENSG00000184922.12 | FMNL1 | 0.797501 | 0.582519 | 1.091823 | 0.158094 |
| PD-1 | ENSG00000134516.14 | DOCK2 | 0.798406 | 0.583176 | 1.093071 | 0.159358 |
| PD-L1 | ENSG00000100767.14 | PAPLN | 0.798889 | 0.583482 | 1.093818 | 0.159513 |
| PD-1 | ENSG00000072818.10 | ACAP1 | 0.800547 | 0.584729 | 1.096022 | 0.165614 |
| PD-1 | ENSG00000188011.5 | RTP5 | 0.801344 | 0.585485 | 1.096787 | 0.166012 |
| PD-1 | ENSG00000010671.14 | BTK | 0.801742 | 0.585126 | 1.09855 | 0.166017 |
| PD-1 | ENSG00000110324.8 | IL10RA | 0.802899 | 0.58659 | 1.098973 | 0.168029 |
| PD-1 | ENSG00000000938.11 | FGR | 0.805881 | 0.588761 | 1.103068 | 0.174015 |
| PD-L1 | ENSG00000134539.15 | KLRD1 | 0.806106 | 0.588961 | 1.103312 | 0.176134 |
| PD-1 | ENSG00000122224.16 | LY9 | 0.813439 | 0.594324 | 1.113336 | 0.19187 |
| PD-1 | ENSG00000139626.14 | ITGB7 | 0.814301 | 0.594164 | 1.115999 | 0.201236 |
| PD-L1 | ENSG00000120217.12 | CD274 | 0.816685 | 0.596641 | 1.117881 | 0.204257 |
| PD-L1 | ENSG00000072786.11 | STK10 | 0.816731 | 0.59671 | 1.11788 | 0.205308 |
| PD-1 | ENSG00000106948.15 | AKNA | 0.818156 | 0.597761 | 1.119811 | 0.206711 |
| PD-L1 | ENSG00000064201.14 | TSPAN32 | 0.820796 | 0.599614 | 1.123566 | 0.216474 |
| PD-1 | ENSG00000122986.12 | HVCN1 | 0.821726 | 0.600074 | 1.12525 | 0.217849 |
| PD-1 | ENSG00000006062.12 | MAP3K14 | 0.821026 | 0.599861 | 1.123733 | 0.217908 |
| PD-1 | ENSG00000242258.1 | LINC00996 | 0.824024 | 0.602024 | 1.127888 | 0.225308 |
| PD-L1 | ENSG00000094963.12 | FMO2 | 0.82471 | 0.601681 | 1.13041 | 0.225937 |
| PD-1 | ENSG00000003400.13 | CASP10 | 0.826059 | 0.603439 | 1.130806 | 0.231172 |
| PD-1 | ENSG00000100365.13 | NCF4 | 0.826914 | 0.604129 | 1.131857 | 0.233721 |
| PD-1 | ENSG00000142347.15 | MYO1F | 0.827505 | 0.604255 | 1.133236 | 0.235508 |
| PD-1 | ENSG00000167984.15 | NLRC3 | 0.830114 | 0.606484 | 1.136205 | 0.241 |
| PD-L1 | ENSG00000186038.8 | HTR3E | 0.828826 | 0.605211 | 1.135063 | 0.244986 |
| PD-1 | ENSG00000158517.12 | NCF1 | 0.831027 | 0.607165 | 1.137428 | 0.247788 |
| PD-1 | ENSG00000013374.14 | NUB1 | 0.833305 | 0.608574 | 1.141023 | 0.251636 |
| PD-1 | ENSG00000185862.6 | EVI2B | 0.833965 | 0.609308 | 1.141457 | 0.252089 |
| PD-L1 | ENSG00000089639.9 | GMIP | 0.831723 | 0.607447 | 1.138805 | 0.252655 |
| PD-L1 | ENSG00000176920.11 | FUT2 | 0.835302 | 0.610044 | 1.143735 | 0.259805 |
| PD-L1 | ENSG00000104903.4 | LYL1 | 0.835688 | 0.609774 | 1.145299 | 0.265124 |
| PD-1 | ENSG00000186517.12 | ARHGAP30 | 0.83874 | 0.61219 | 1.149129 | 0.272706 |
| PD-L1 | ENSG00000091490.9 | SEL1L3 | 0.844776 | 0.617017 | 1.156608 | 0.290781 |
| PD-1 | ENSG00000115956.9 | PLEK | 0.846036 | 0.61804 | 1.158141 | 0.292518 |
| PD-L1 | ENSG00000236751.1 | LINC01186 | 0.845601 | 0.617334 | 1.158273 | 0.293 |
| PD-1 | ENSG00000083454.20 | P2RX5 | 0.84582 | 0.617784 | 1.158027 | 0.293913 |
| PD-1 | ENSG00000180549.7 | FUT7 | 0.847114 | 0.618823 | 1.159624 | 0.297544 |
| PD-1 | ENSG00000008517.15 | IL32 | 0.847844 | 0.619274 | 1.16078 | 0.300669 |
| PD-1 | ENSG00000146192.13 | FGD2 | 0.847944 | 0.619023 | 1.161524 | 0.301859 |
| PD-L1 | ENSG00000108950.10 | FAM20A | 0.850027 | 0.621029 | 1.163467 | 0.307354 |
| PD-1 | ENSG00000079263.17 | SP140 | 0.851364 | 0.622033 | 1.165246 | 0.310961 |
| PD-1 | ENSG00000172578.10 | KLHL6 | 0.861722 | 0.629151 | 1.180265 | 0.347107 |
| PD-L1 | ENSG00000100599.14 | RIN3 | 0.862636 | 0.630268 | 1.180673 | 0.353062 |
| PD-L1 | ENSG00000234199.2 | LINC01191 | 0.86402 | 0.631058 | 1.182983 | 0.360017 |
| PD-1 | ENSG00000065413.15 | ANKRD44 | 1.15412 | 0.842557 | 1.580893 | 0.362449 |
| PD-L1 | ENSG00000026950.15 | BTN3A1 | 0.865365 | 0.63217 | 1.18458 | 0.364969 |
| PD-L1 | ENSG00000049768.13 | FOXP3 | 0.866805 | 0.633207 | 1.186579 | 0.371171 |
| PD-1 | ENSG00000184293.6 | CLECL1 | 0.867766 | 0.634014 | 1.187699 | 0.372642 |
| PD-1 | ENSG00000126882.11 | FAM78A | 0.869742 | 0.634736 | 1.191757 | 0.382547 |
| PD-1 | ENSG00000115935.15 | WIPF1 | 0.872338 | 0.637311 | 1.194038 | 0.389451 |
| PD-L1 | ENSG00000076641.4 | PAG1 | 0.871991 | 0.636986 | 1.193697 | 0.391874 |
| PD-1 | ENSG00000140968.9 | IRF8 | 0.873654 | 0.637942 | 1.196458 | 0.393753 |
| PD-L1 | ENSG00000075891.20 | PAX2 | 1.144568 | 0.835411 | 1.568133 | 0.39396 |
| PD-1 | ENSG00000124196.5 | GTSF1L | 0.874588 | 0.639001 | 1.197031 | 0.401385 |
| PD-1 | ENSG00000227145.1 | IL21-AS1 | 0.876367 | 0.640302 | 1.199465 | 0.407662 |
| PD-L1 | ENSG00000085265.9 | FCN1 | 0.8782 | 0.641578 | 1.202092 | 0.411983 |
| PD-L1 | ENSG00000104951.14 | IL4I1 | 0.87786 | 0.641249 | 1.201778 | 0.415093 |
| PD-1 | ENSG00000189233.10 | NUGGC | 0.878698 | 0.64194 | 1.202776 | 0.415989 |
| PD-1 | ENSG00000278195.1 | SSTR3 | 0.88158 | 0.644037 | 1.206738 | 0.430934 |
| PD-L1 | ENSG00000057657.13 | PRDM1 | 0.884 | 0.645706 | 1.210235 | 0.43495 |
| PD-L1 | ENSG00000101445.8 | PPP1R16B | 0.883659 | 0.645574 | 1.209548 | 0.436021 |
| PD-L1 | ENSG00000101342.8 | TLDC2 | 0.885295 | 0.646518 | 1.212259 | 0.444746 |
| PD-L1 | ENSG00000100100.11 | PIK3IP1 | 0.886726 | 0.647869 | 1.213646 | 0.447219 |
| PD-L1 | ENSG00000104880.16 | ARHGEF18 | 0.887049 | 0.647622 | 1.214991 | 0.455102 |
| PD-L1 | ENSG00000102057.8 | KCND1 | 0.889893 | 0.65018 | 1.217987 | 0.4627 |
| PD-L1 | ENSG00000100368.12 | CSF2RB | 0.892942 | 0.652298 | 1.222364 | 0.473953 |
| PD-1 | ENSG00000128815.16 | WDFY4 | 0.894203 | 0.653214 | 1.224099 | 0.477417 |
| PD-L1 | ENSG00000048740.16 | CELF2 | 0.895207 | 0.654065 | 1.225253 | 0.48638 |
| PD-1 | ENSG00000213203.2 | GIMAP1 | 0.901494 | 0.65839 | 1.23436 | 0.513469 |
| PD-1 | ENSG00000196329.9 | GIMAP5 | 0.912519 | 0.666374 | 1.249585 | 0.564722 |
| PD-L1 | ENSG00000103472.8 | RRN3P2 | 0.915166 | 0.668646 | 1.252574 | 0.574891 |
| PD-1 | ENSG00000204165.5 | CXorf65 | 0.916141 | 0.669328 | 1.253967 | 0.580086 |
| PD-L1 | ENSG00000101265.14 | RASSF2 | 0.916638 | 0.669354 | 1.255278 | 0.583019 |
| PD-L1 | ENSG00000081052.11 | COL4A4 | 0.9225 | 0.673865 | 1.262873 | 0.612238 |
| PD-L1 | ENSG00000177807.6 | KCNJ10 | 0.923935 | 0.674981 | 1.264712 | 0.618357 |
| PD-L1 | ENSG00000188263.9 | IL17REL | 0.926683 | 0.677045 | 1.268365 | 0.634068 |
| PD-1 | ENSG00000069424.13 | KCNAB2 | 0.92813 | 0.677464 | 1.271544 | 0.639317 |
| PD-L1 | ENSG00000083457.10 | ITGAE | 1.07471 | 0.784923 | 1.471482 | 0.650058 |
| PD-1 | ENSG00000141968.6 | VAV1 | 0.931608 | 0.680176 | 1.275984 | 0.656412 |
| PD-L1 | ENSG00000091592.14 | NLRP1 | 0.932378 | 0.680732 | 1.277049 | 0.659768 |
| PD-L1 | ENSG00000253187.2 | HOXA10-AS | 0.932624 | 0.68091 | 1.277391 | 0.662694 |
| PD-L1 | ENSG00000089692.7 | LAG3 | 0.933725 | 0.682114 | 1.278149 | 0.668052 |
| PD-L1 | ENSG00000145358.5 | DDIT4L | 1.069318 | 0.781162 | 1.463768 | 0.675069 |
| PD-L1 | ENSG00000137757.9 | CASP5 | 1.067793 | 0.779541 | 1.46263 | 0.681269 |
| PD-L1 | ENSG00000092067.5 | CEBPE | 1.065634 | 0.776369 | 1.462675 | 0.691288 |
| PD-1 | ENSG00000182487.11 | NCF1B | 0.94263 | 0.68791 | 1.291669 | 0.711949 |
| PD-L1 | ENSG00000081320.9 | STK17B | 1.059609 | 0.773903 | 1.450791 | 0.717067 |
| PD-1 | ENSG00000103522.14 | IL21R | 0.949721 | 0.69361 | 1.300399 | 0.746725 |
| PD-1 | ENSG00000137841.10 | PLCB2 | 0.951721 | 0.694174 | 1.304822 | 0.755522 |
| PD-L1 | ENSG00000100060.16 | MFNG | 0.954562 | 0.69725 | 1.306831 | 0.76713 |
| PD-1 | ENSG00000137101.11 | CD72 | 0.954026 | 0.695827 | 1.308034 | 0.768142 |
| PD-1 | ENSG00000138684.6 | IL21 | 0.953926 | 0.695436 | 1.308496 | 0.770042 |
| PD-L1 | ENSG00000205929.8 | C21orf62 | 1.045524 | 0.763792 | 1.431175 | 0.780045 |
| PD-L1 | ENSG00000090659.16 | CD209 | 1.044664 | 0.761767 | 1.432621 | 0.782625 |
| PD-L1 | ENSG00000159921.13 | GNE | 0.95835 | 0.700169 | 1.311734 | 0.789763 |
| PD-L1 | ENSG00000049249.7 | TNFRSF9 | 0.960877 | 0.702009 | 1.315202 | 0.800852 |
| PD-L1 | ENSG00000105464.3 | GRIN2D | 1.0339 | 0.755297 | 1.41527 | 0.834615 |
| PD-L1 | ENSG00000152207.6 | CYSLTR2 | 0.967985 | 0.707104 | 1.325115 | 0.836147 |
| PD-L1 | ENSG00000084070.10 | SMAP2 | 1.032745 | 0.754204 | 1.414157 | 0.839178 |
| PD-L1 | ENSG00000160326.12 | SLC2A6 | 1.032153 | 0.754063 | 1.412799 | 0.843038 |
| PD-1 | ENSG00000010295.18 | IFFO1 | 0.971825 | 0.709171 | 1.331758 | 0.857573 |
| PD-L1 | ENSG00000162897.13 | FCAMR | 1.028659 | 0.751501 | 1.408034 | 0.859797 |
| PD-1 | ENSG00000007129.16 | CEACAM21 | 0.975283 | 0.712573 | 1.334848 | 0.875312 |
| PD-L1 | ENSG00000068831.17 | RASGRP2 | 1.018252 | 0.743827 | 1.393922 | 0.909275 |
| PD-L1 | ENSG00000198785.4 | GRIN3A | 1.017859 | 0.743626 | 1.393224 | 0.911497 |
| PD-L1 | ENSG00000250328.4 | MGC32805 | 1.008094 | 0.736492 | 1.379857 | 0.959794 |
| PD-L1 | ENSG00000101096.18 | NFATC2 | 1.005551 | 0.73435 | 1.376908 | 0.97184 |
| PD-L1 | ENSG00000077238.12 | IL4R | 1.002533 | 0.732482 | 1.372145 | 0.987297 |
| PD-L1 | ENSG00000071246.9 | VASH1 | 0.998244 | 0.728684 | 1.367521 | 0.991207 |
